# A Systematic Review of the Role of Diet in Ulcerative Colitis

**DOI:** 10.7759/cureus.39350

**Published:** 2023-05-22

**Authors:** Nasir Abbas, Mahrukh Shakil, Zeeshan Akhtar Rana, Sardar Basharat Ali, Ammad Ayub Awan, Saman Gul

**Affiliations:** 1 Trauma and Emergency, Combined Military Hospital, Sialkot, PAK; 2 Internal Medicine, Combined Military Hospital, Sialkot, PAK; 3 Internal Medicine, Jinnah Social Welfare Hospital, Sialkot, PAK; 4 Internal Medicine, Khan Clinic and Maternity Home, Lahore, PAK; 5 Internal Medicine, District Headquarter Hospital, Khushab, PAK; 6 Internal Medicine, Niazi Medical and Dental College, Sargodha, PAK

**Keywords:** management, evidence-based, randomized controlled trials, clinical trials, diet, ulcerative colitis

## Abstract

Ulcerative colitis (UC) is a chronic inflammatory bowel disease characterized by inflammation and ulceration of the colon and rectum. Diet is an important part of UC management because it can either aggravate or alleviate symptoms along with medication therapy. A comprehensive literature search was conducted using multiple databases (PubMed and Online Wiley Library) and search engines (Google Scholar) using specific keywords related to UC and diet. The search resulted in a large number of articles, which were then narrowed down by focusing on clinical trials and randomized controlled trials published between 2010 and 2023. According to the research, certain dietary interventions, such as the low FODMAP diet, the Mediterranean diet, and the anti-inflammatory diet, appear to improve symptoms and overall quality of life. Dietary interventions have the potential to help with UC management. The goal should be to provide patients with tailored dietary interventions and other treatments to improve their quality of life. More research is needed to identify the most effective dietary interventions and better understand how they work.

## Introduction and background

Ulcerative colitis (UC) is a chronic inflammatory disease of the colon that is characterized by abdominal pain, rectal bleeding, and diarrhea [1,2]. Research suggests that a combination of genetic, environmental, and immune factors may be involved in the development of UC; however, the exact cause of the condition remains unknown [3,4]. It has also been recognized that diet may play a role in the development of UC as well as its treatment. It is essential for a variety of reasons to have a good understanding of the role that diet plays in the treatment of UC. Dietary interventions have the potential to provide a treatment option that is both safe and effective as an alternative or complement to conventional medication [5,6]. Patients diagnosed with UC can improve their health by making changes to their diet as diet is a modifiable risk factor. Healthcare providers can increase patient engagement and promote self-management by giving patients the authority to make dietary improvements. Studies suggest that certain dietary factors may be associated with a lower or higher risk of developing UC. This complexity and multifactorial nature of the influence of diet on UC is reflected in the findings of these studies. In addition, dietary interventions such as exclusive enteral nutrition or exclusion diets have been shown to alleviate symptoms of UC and induce remission in patients with UC [7-9]. To develop effective treatment strategies and improve patient outcomes, it is essential to have a good understanding of the association between diet and UC.

## Review

Methodology

To investigate the relationship between UC and diet, we conducted a comprehensive literature search utilizing multiple databases, such as PubMed and Wiley Online Library. The literature was also searched using Google Scholar. We utilized specific keywords and phrases related to UC and diet to identify relevant studies from databases and search engines, such as ulcerative colitis diet, PubMed: (ulcerative colitis[Title/Abstract]) AND (diet[Title/Abstract]), PubMed: (ulcerative colitis[Title]) AND (diet[Title]), PubMed: (ulcerative colitis[Title/Abstract]) AND (diet[Title/Abstract]) AND ((clinicaltrial[Filter] OR randomizedcontrolledtrial[Filter]) AND (2010:2023[pdat])) and Wiley Online Library: “ulcerative colitis” in Title and “diet” in Title.

**Figure 1:**
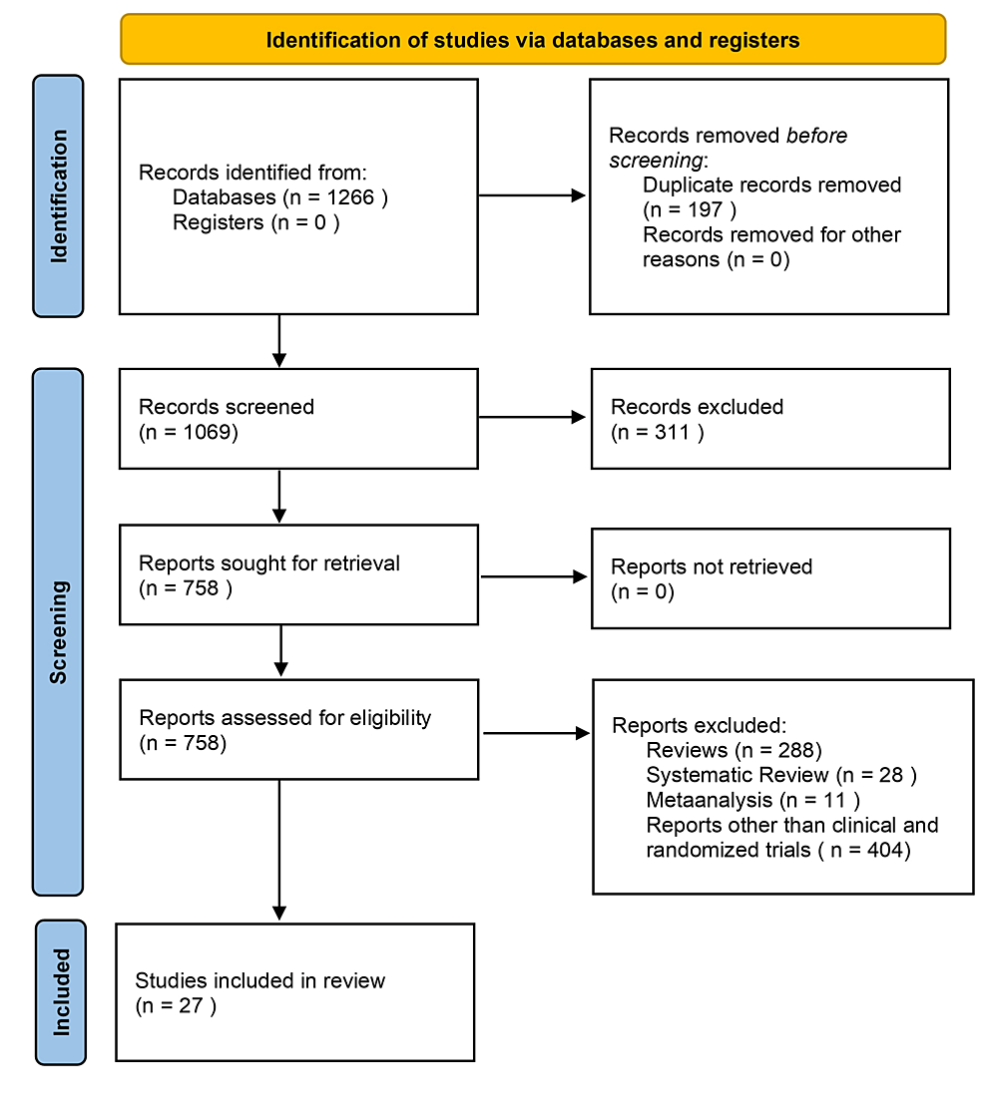
Preferred Reporting Items for Systematic Reviews and Meta-Analyses flow diagram employed for this review.

The initial search yielded a total of 1,266 articles, 1,069 articles from PubMed, 185 articles from Google Scholar, and 12 articles from Wiley Online Library. After removing duplicate records (n = 197), 1,069 articles remained for screening. During the screening process, 311 articles were excluded due to reasons such as not addressing the research question, language limitations, and lack of rigorous peer review (conference abstracts). Following this, articles were sought for retrieval and assessed for eligibility, resulting in 758 articles. From these, several types of articles were excluded, including 288 reviews, 28 systematic reviews, 11 meta-analyses, and 404 articles that were not clinical or randomized trials. Subsequently, we narrowed our search by focusing on clinical trials and randomized controlled trials that investigated the impact of diet on UC as they provide the highest level of evidence. This resulted in a total of 27 articles published between 2010 and 2023 that met our inclusion criteria (clinical trials and randomized controlled trials involving participants diagnosed with UC; articles evaluating the impact of dietary interventions on UC, which included various dietary approaches, such as specific dietary patterns, supplementation, exclusion diets, or any other dietary interventions relevant to the management of UC; and studies reporting relevant outcomes related to UC, including disease activity scores, clinical remission rates, endoscopic findings, histological changes, quality of life measures, or any other clinically relevant parameters associated with the impact of diet on UC). Specific exclusion criteria were implemented to ensure the selection of appropriate studies for analysis. Review articles were excluded as the focus was on original research presenting primary data rather than a summary or interpretive works. Systematic review articles and meta-analyses were excluded to avoid duplication of efforts and potential bias in the synthesis of evidence. Reports and studies other than clinical trials and randomized controlled trials were excluded to prioritize studies with the highest level of evidence. This involved excluding case reports, case series, observational studies, and other non-experimental study designs. Figure 1 illustrates the Preferred Reporting Items for Systematic Reviews and Meta-Analyses flow diagram demonstrating the search process for this review.

Results

Our search strategy allowed us to obtain a comprehensive view of the literature on this subject from various sources and viewpoints (Table 1). By carefully examining the results of these studies, we hope to shed light on the safest and most effective dietary approaches for patients with UC.

**Table 1:** Studies on the relationship between diet and ulcerative colitis. UC: ulcerative colitis; FMT: fecal microbiota transplantation; FMT-AID: FMT with an anti-inflammatory diet; SMT: standard medical therapy; FT: Fecal transplantation; SCFAs: short-chain fatty acids; FR-QoL: food-related quality of life; MSCD: modified specific carbohydrate diet; SCD: specific carbohydrate diet; IBD: inflammatory bowel disease; AID: anti-inflammatory diet; PBD: plant-based diet; Md: Mediterranean diet; CD: Crohn’s disease; FGS: functional gastrointestinal symptoms; GOS: galacto-oligosaccharides; LFD: low-fat, high-fiber diet; iSAD: improved standard American diet; LESS: laparoendoscopic single-site; SCCAI: Simple Clinical Colitis Activity Index; IBS: irritable bowel syndrome; EVOO: extra virgin olive oil; PAA: phenylacetic acid; 4-HPAA: 4-hydroxyphenylacetic acid; IAA: 3-indolylacetic acid; SA: succinic acid; FA: fumaric acid; PPA: 3-phenylpropionic acid; IPA: 3-indolepropionic acid; CMP: cow’s milk protein

Author/Year	Study	Participants	Findings
Kedia et al., (2022) [5]	To investigate the effectiveness of multi-donor FMT and an anti-inflammatory diet in causing remission, followed by long-term maintenance with an anti-inflammatory diet in patients with mild-to-moderate UC	66 patients were randomized to receive either multi-donor FMT-AID or optimized SMT	At 8 weeks, FMT-AID outperformed SMT in terms of inducing clinical response, remission, and deep remission. An anti-inflammatory diet outperformed SMT in sustaining deep remission for 48 weeks
Sarbagili Shabat et al., (2022) [6]	FT remission rate in refractory UC and the effect of novel diets for donors and patients	62 patients with refractory UC	UCED seemed to achieve greater clinical remission and mucosal healing than single-donor FT with or without diet
Day et al., (2022) [7]	The impact of the 4-SURE diet on UC patients with mild-to-moderate activity	28 adults with UC	The 4-SURE diet was well tolerated, and 46% and 36% of participants improved clinically and endoscopically, respectively. SCFA excretion increased by 69%, while the proportion of branched-chain fatty acids to SCFAs decreased by 27%. FRQoL was also improved by the diet
Kaplan et al., (2022) [8]	Effects of MSCD and SCD on the symptoms and inflammation in patients with IBD	54 patients aged 7–18 years with IBD and active inflammation	Neither SCD nor MSCD consistently improved symptoms or inflammation. Some people improved their symptoms and fecal calprotectin levels when compared to their UD, while others did not
Keshteli et al., (2022) [9]	Patients with UC and the effects of an AID on subclinical inflammation	53 adult UC patients in clinical remission	Compared to the control group, the AID group had a lower relapse rate and a higher subclinical response rate. Changes in the metabolome and microbiota in the AID group were also associated with a decrease in inflammation
Cabrera-Acosta et al., (2012) [10]	The ability of patients with chronic idiopathic UC to digest lactose and their lactose intolerance	39 patients with chronic idiopathic UC	Lactose digestion was impaired in 46% of patients with chronic idiopathic UC. There were no differences in symptoms, duration, or progression of chronic idiopathic UC between patients who could and could not digest lactose
Chiba et al., (2019) [11]	Ulcerative colitis relapse can be prevented by following a PBD	92 patients with UC	In patients with UC, a PBD was associated with a lower relapse rate than conventional therapy. At one and five-year follow-ups, the cumulative relapse rates for initial episode cases were 14% and 27%, respectively, and 36% and 53%, respectively, for relapse cases. Even after a six-year follow-up, PBD adherence was significantly higher than at baseline
Chiba et al., (2020) [12]	When treating severe UC, the effectiveness of a PBD and infliximab as first-line treatments	17 patients with severe UC	In the induction phase, a PBD and infliximab as first-line therapy for severe UC resulted in a 76% remission rate and a 6% colectomy rate. C-reactive protein levels and erythrocyte sedimentation rate both decreased significantly by week six. At one year, the cumulative relapse rate was 25%, with no additional colectomy cases. PBD mean scores of 27.7 after one year and 23.8 after four years were significantly higher than baseline scores of 8.3 and 9.9, respectively
Chicco et al., (2021) [13]	The effects of Md on patients with IBD in terms of their nutritional status, liver steatosis, clinical disease activity, and QoL	142 IBD patients, 84 UC patients, and 58 CD patients	IBD patients benefit from Md. It can lower body mass index and waist circumference, decrease the number of patients with liver steatosis, decrease the number of IBD patients with active disease, and improve QoL
Cox et al., (2017) [14]	To ascertain whether fermentable carbohydrates worsen FGS in IBD	32 IBD patients who met the criteria for functional gas, bloating or diarrhea. Data were available for 29 patients who completed all arms (12 with CD and 17 with UC)	Fructans, but not GOS or sorbitol, exacerbated FGS in quiescent IBD at relatively high doses
Cox et al., (2020) [15]	The effects of a low FODMAP diet on persistent digestive symptoms, the intestinal microbiome, and circulating inflammatory markers in patients with dormant IBD	52 patients with quiescent CD or UC and persisting gastrointestinal symptoms	The low FODMAP diet resulted in a higher proportion of patients reporting adequate relief of gut symptoms than the control diet. Following the low FODMAP diet, patients had higher health-related QoL scores. When compared to the control diet, the low FODMAP diet reduced the fecal abundance of microbes thought to regulate the immune response
Cox et al., (2022) [16]	Studying the feasibility trial of improving FR-QoL in newly diagnosed IBD patients by using a web resource	50 participants were randomized, 30 to the web resource and 20 to control	When compared to the control group, the web resource group had a significant improvement in FR-QoL and IBD distress. At the end of the trial, the web resource group also had lower CD clinical activity
Fritsch et al., (2021) [17]	To evaluate the effects of an LFD and an iSAD on fecal markers of intestinal dysbiosis, markers of inflammation, and QoL in patients with UC in remission	A four-week period of either an LFD or an iSAD treatment, followed by a two-week washout period, was given to 17 patients with UC who were in remission or had a mild disease (with a flare within the previous 18 months)	Both diets were tolerated well and improved QoL. However, the LFD reduced inflammation markers and intestinal dysbiosis in fecal samples
Gash et al., (2011) [18]	To examine the viability and safety of LESS colorectal resection using conventional instrumentation	20 consecutive unselected patients	When performed by an experienced team, LESS colorectal resection using conventional instrumentation was feasible and safe. The LESS approach may offer benefits such as reduced pain, improved cosmesis, lower costs, and faster recovery
Grimstad et al., (2011) [19]	A diet high in salmon may help treat mild UC	12 patients with mild UC	The consumption of 600 g of Atlantic salmon weekly for eight weeks resulted in a significant decrease in the SCCAI as well as an increase in n-3 polyunsaturated fatty acid concentrations in plasma and rectal biopsies. In both biopsies and plasma, the anti-inflammatory fatty acid index increased
James et al., (2015) [20]	Patients with UC in remission and the impact of dietary fiber on colonic fermentation	10 healthy controls and 19 UC patients	When compared to controls, UC patients had lower proportions of Akkermansia muciniphila and higher diversity within Clostridium cluster XIVa. The gut fermentation of NSP and starch was reduced in UC patients, which could not be explained by abnormal gut transit. In UC patients, increasing RS/WB intake did not increase the proportion of NSP fermented, but it did normalize gut transit
Jia et al., (2010) [21]	Patients with gut diseases and healthy controls’ responses to treatment and the prevalence of F. prausnitzii	10 healthy volunteers, 15 patients with active CD, 15 patients with UC, 15 patients with IBS	At the time of presentation, F. prausnitzii levels were lower in CD patients than in healthy controls and UC patients. Elemental diet treatment reduced the Harvey and Bradshaw index as well as serum C-reactive protein concentrations in all groups. After treatment, F. prausnitzii levels decreased significantly in all groups, but the decrease was more pronounced in CD patients
Jian et al., (2018) [22]	The efficacy of an IgG-guided exclusion diet in UC patients	97 patients with UC were randomly divided into an intervention group	When compared to the control group, the intervention group, which followed a standard diet, experienced a significant improvement in UC symptoms and QoL
Krag et al., (2012) [23]	Patients with active UC can be put into remission with Profermin(®) with minimal risk	39 patients with mild-to-moderate UC	Profermin(®) was well tolerated and reduced the SCCAI score significantly. Sixty-two percent of patients met the primary endpoint of a 50% reduction in SCCAI, and 46% met the secondary endpoint of remission (SCCAI 2.5)
Kyaw et al., (2014) [24]	The effects of comprehensive dietary recommendations on the clinical course of the illness and quality of life in UC patients	112 patients with UC	Patients who adhered to strict dietary guidelines saw significant improvements in their symptoms and quality of life
Limdi et al., (2016) [25]	Dietary attitudes and practices of patients with IBD	400 consecutive IBD patients attending the IBD clinics	The majority of patients thought diet could be an initiating factor or trigger for an IBD flare. They also reported that certain foods aggravated their symptoms and that they had to deprive themselves of their favorite foods to avoid relapse. Many patients also believed that IBD had a negative impact on their appetite and that they had never received formal dietary advice
Melgaard et al., (2022) [26]	The effects of a low FODMAP diet on IBD and IBS patients’ symptoms and quality of life	19 patients with IBD and IBS symptoms	Eliminating low FODMAP foods for two weeks reduced pain and bloating significantly. After two weeks of double-blind provocations with a placebo, pain and bloating scores returned to baseline
Morvaridi et al., (2020) [27]	Treatment of UC and EVOO	40 patients with UC	When compared to canola oil, patients who consumed EVOO had a significant decrease in inflammatory markers and improved gastrointestinal symptoms
Racine et al., (2016) [28]	Dietary factors and the risk of UC	366,351 participants with IBD, including 256 incident cases of UC and 117 incident cases of CD, and four matched controls per case	A diet imbalance characterized by a high intake of sugar and soft drinks and a low intake of vegetables was linked to an increased risk of UC
Sánchez-Morales et al., (2019) [29]	The impact of probiotics on patients with UC in terms of histological changes, clinical changes, and ability to tolerate food	17 patients with mild-to-moderate UC in each group	Patients who received a combination of six probiotic strains improved significantly in terms of symptoms, histological findings, and feeding tolerance when compared to those who did not receive probiotics
Sitkin et al., (2013) [30]	UC and CD patients with metabolomics-based serum metabolomic profiles	75 people were involved in the study: 20 patients with mild-to-moderate active UC, 35 CD patients, and 20 healthy controls	UC patients had significantly higher levels of PAA, 4-HPAA, IAA, SA, and FA, but significantly lower levels of PPA. IAA, IPA, SA, and FA levels were significantly higher in CD patients
Strisciuglio et al., (2013) [31]	The effectiveness of a CMP elimination diet on initiating and maintaining remission in young patients with UC	29 children with newly diagnosed UC	In children with UC, a CMP elimination diet was not associated with a better response to induction therapy or a lower relapse rate than a free diet

Discussion

Over the past few years, there has been a rise in awareness regarding the importance of diet in the treatment of UC. Dietary interventions have been shown to have positive effects on the symptoms of inflammatory bowel disease (IBD), as well as on the overall quality of life [32,33]. Numerous studies have examined the impact of various diets on UC and its various symptoms, signs, and complications. In a study by Kedia et al., the effectiveness of multi-donor fecal microbiota transplantation (FMT) and an anti-inflammatory diet in inducing and maintaining remission in patients with mild-to-moderate UC was examined [5]. Sarbagili Shabat et al. discovered that in terms of producing a clinical response, remission, and deep remission, FMT in combination with an anti-inflammatory diet outperformed standard medical therapy. In patients with refractory UC, fecal transplantation (FT) was compared with novel diets for donors and patients [6]. In comparison to single-donor FT with or without diet, the authors discovered that FT combined with a novel diet (UCED) led to greater clinical remission and mucosal healing. Day et al. examined the effects of the 4-SURE diet on UC patients with mild-to-moderate activity and discovered that a sizable portion of participants experienced improvements in their clinical and endoscopic conditions as a result of the diet [7]. Kaplan et al. examined how the modified specific carbohydrate diet (MSCD) and the specific carbohydrate diet (SCD) affected symptoms and inflammation in IBD patients and discovered that the effects were inconsistent [8]. Chiba et al. investigated how a plant-based diet (PBD) affected UC patients and discovered that it was linked to a lower relapse rate when compared to conventional therapy. According to Chiba et al., using a PBD and infliximab as first-line therapy in severe UC cases led to a high rate of remission and a low rate of colectomy [11,12]. The Mediterranean diet which reduced liver steatosis, clinical disease activity, and quality of life for IBD patients, including those with UC, was also found to be helpful [13]. The impact of dietary components and interventions on UC has been examined in other studies. For instance, Cox et al. (2017) discovered that fructans made quiescent UC patients’ functional gastrointestinal symptoms worse. However, Cox et al. (2020) demonstrated that in patients with dormant IBD, a low FODMAP diet decreased persistent digestive symptoms and enhanced the intestinal microbiome and inflammatory markers. In patients with IBD and irritable bowel syndrome (IBS) symptoms, Melgaard et al. (2022) showed that removing low FODMAP foods significantly reduced pain and bloating [14,15,26].

It has also been discovered that the Mediterranean diet is beneficial for IBD patients by reducing liver steatosis, active IBD, body mass index (BMI), and waist circumference, as well as by enhancing their quality of life [13,34]. The 4-SURE diet was well tolerated, and clinical and endoscopic conditions improved in 46% and 36% of participants, respectively [7]. By reducing intestinal dysbiosis and inflammation markers, both a low-fat, high-fiber diet (LFD) and an improved standard American diet (iSAD) improved the quality of life in UC patients in remission [17]. However, neither the SCD nor the MSCD consistently reduced UC patients’ symptoms or inflammation [8].

In contrast, the autoimmune protocol diet (AID) had a higher subclinical response rate and a lower relapse rate than the control group, which may have been due to changes in the AID group’s metabolome and microbiome that reduced inflammation [5]. According to another study, adhering to a strict diet that includes fruits, vegetables, and whole grains while limiting red meat, processed foods, and dairy products can help reduce symptoms and improve the quality of life in patients with UC [35-37]. Many IBD patients believe that their diet causes flare-ups and avoid their favorite foods as a result.

A study found that limiting FODMAP foods reduced IBD and IBS patients’ discomfort and bloating. Moreover, the low FODMAP diet reduced gut symptoms in a greater proportion of UC patients than in the control group [15,26,38,39]. It was discovered that extra virgin olive oil reduces inflammatory markers and improves gastrointestinal symptoms. However, a diet high in sugar and soft drinks and low in vegetables increases the likelihood of developing UC. In addition, patients who received six probiotic strains exhibited improved symptoms, histological findings, and feeding tolerance [27].

UC patients had higher levels of phenylacetic acid, 4-hydroxyphenylacetic acid, 3-indolylacetic acid (IAA), succinic acid (SA), and fumaric acid (FA), whereas Crohn’s disease (CD) patients had lower levels of 3-phenylpropanoic acid. Patients with CD had elevated levels of IAA, 3-indolepropionic acid, SA, and FA [30]. A cow’s milk protein elimination diet did not affect induction therapy response or relapse rate in newly diagnosed UC children [31]. A recent study found that FMT when combined with an anti-inflammatory diet was superior to standard medical therapy in this regard. However, it is essential to keep in mind that FMT is still regarded as an experimental treatment, and, as such, it ought to be performed only under the supervision of a qualified medical expert [5,40-42].

Several review studies have also been conducted on this subject. A high-fat diet can cause UC by disrupting the intestinal mucosal barrier, causing dysbiosis, reducing goblet and Paneth cell secretion, and impairing intercellular interactions, according to Jiang et al. Due to gut metabolites that stimulate proinflammatory pathways and reduce anti-inflammatory immune cell effects, the disruption can also result in an intestinal immune imbalance. A study investigated the molecular mechanisms and potential dietary interventions for treating and preventing UC caused by a disrupted intestinal mucosal barrier [43].

Sinopoulou et al. discussed how to manage abdominal pain in patients with UC and how it can be a sign of disease relapse or other complications. The authors conducted a systematic review of interventions for managing abdominal pain in UC patients and discovered that no conclusions about the effectiveness of any of the interventions could be drawn due to very low certainty of evidence. The low certainty was due to a lack of data and the possibility of bias. The Grading of Recommendations, Assessment, Development, and Evaluations methodology was also used by the authors to assess the certainty of evidence [44].

According to Yao et al. and Teigen et al., dietary therapies that target the microbial production of gaseous metabolites, specifically hydrogen sulfide and nitric oxide, may reduce mucosal inflammation in UC. As potential interventions, the authors propose a low-sulfur diet or the use of dietary supplements to inhibit the production of these gases. The authors discuss how decreasing H_2_S production in the gut may result in less mucosal inflammation. However, the efficacy of this dietary intervention is limited, and more research is needed to determine its safety and efficacy [45,46].

Radziszewska et al. and LeBlanc et al. discovered no compelling evidence to support any specific dietary prescription for improving clinical outcomes. However, they did note that a Mediterranean diet may be beneficial for UC. Other dietary interventions, such as a low-residue diet, exclusion diets, or specific carbohydrate diets, may also play a role in UC management, but more research is needed to determine their effectiveness [47,48].

In general, the findings of these studies point to the possibility that dietary modifications can play a part in the management of UC. However, it is essential to keep in mind that dietary interventions should be tailored to the individual patient following his/her specific requirements and should be performed under supervision.

Limitations

Our search strategy may have been subject to limitations as we used a limited set of keywords and focused on PubMed as the primary database. This approach may have resulted in potentially missing relevant studies from other databases such as Google Scholar. Additionally, our inclusion and exclusion criteria might have inadvertently excluded relevant studies. Therefore, the potential for publication bias and the exclusion of important studies should be considered.

## Conclusions

Patients with UC may be able to improve their symptoms and overall quality of life through dietary interventions. While FODMAP-restricted diets, the Mediterranean diet, and anti-inflammatory diets have shown promise in reducing intestinal inflammation and improving UC patient outcomes, other types of diets mentioned in previous studies should also be considered. Multi-donor FMT-AID, the 4-SURE diet, MSCD, AID, a PBD, and LFD are some examples. In addition, the effectiveness of an IgG-guided exclusion diet, Profermin(®), comprehensive dietary recommendations, and a low FODMAP diet has been examined. It is important to note that the efficacy of these diets can vary from person to person and that some diets may have negative effects on nutrition, necessitating careful monitoring. To better comprehend the mechanisms and long-term effects of various dietary interventions on UC, additional research is required.

## References

[1] Connor SJ, Sechi A, Andrade M, Deuring JJ, Witcombe D (2021). Ulcerative colitis narrative findings: Australian survey data comparing patient and physician disease management views. JGH Open.

[2] Scribano ML, Papi C, Costa F (2019). Management of ulcerative colitis in a real-life setting: an Italian multicenter, prospective, observational AIGO study. Dig Liver Dis.

[3] Marinelli C, Zingone F, Inferrera M (2020). Factors associated with disability in patients with ulcerative colitis: a cross-sectional study. J Dig Dis.

[4] Hata K, Ishihara S, Nozawa H (2017). Pouchitis after ileal pouch-anal anastomosis in ulcerative colitis: diagnosis, management, risk factors, and incidence. Dig Endosc.

[5] Kedia S, Virmani S, K Vuyyuru S (2022). Faecal microbiota transplantation with anti-inflammatory diet (FMT-AID) followed by anti-inflammatory diet alone is effective in inducing and maintaining remission over 1 year in mild to moderate ulcerative colitis: a randomised controlled trial. Gut.

[6] Sarbagili Shabat C, Scaldaferri F, Zittan E (2022). Use of faecal transplantation with a novel diet for mild to moderate active ulcerative colitis: the CRAFT UC randomised controlled trial. J Crohns Colitis.

[7] Day AS, Yao CK, Costello SP, Ruszkiewicz A, Andrews JM, Gibson PR, Bryant RV (2022). Therapeutic potential of the 4 strategies to SUlfide-REduction (4-SURE) diet in adults with mild to moderately active ulcerative colitis: an open-label feasibility study. J Nutr.

[8] Kaplan HC, Opipari-Arrigan L, Yang J (2022). Personalized research on diet in ulcerative colitis and Crohn's disease: a series of N-of-1 diet trials. Am J Gastroenterol.

[9] Keshteli AH, Valcheva R, Nickurak C (2022). Anti-inflammatory diet prevents subclinical colonic inflammation and alters metabolomic profile of ulcerative colitis patients in clinical remission. Nutrients.

[10] Cabrera-Acosta GA, Milke-García MP, Ramírez-Iglesias MT, Uscanga L (2012). [Deficient lactose digestion and intolerance in a group of patients with chronic nonspecific ulcerative colitis: a controlled, double-blind, cross-over clinical trial]. Rev Gastroenterol Mex.

[11] Chiba M, Nakane K, Tsuji T (2019). Relapse prevention by plant-based diet incorporated into induction therapy for ulcerative colitis: a single-group trial. Perm J.

[12] Chiba M, Tsuji T, Nakane K (2020). High remission rate with infliximab and plant-based diet as first-line (IPF) therapy for severe ulcerative colitis: single-group trial. Perm J.

[13] Chicco F, Magrì S, Cingolani A (2021). Multidimensional impact of Mediterranean diet on IBD patients. Inflamm Bowel Dis.

[14] Cox SR, Prince AC, Myers CE, Irving PM, Lindsay JO, Lomer MC, Whelan K (2017). Fermentable carbohydrates [FODMAPs] exacerbate functional gastrointestinal symptoms in patients with inflammatory bowel disease: a randomised, double-blind, placebo-controlled, cross-over, re-challenge trial. J Crohns Colitis.

[15] Cox SR, Lindsay JO, Fromentin S (2020). Effects of low FODMAP diet on symptoms, fecal microbiome, and markers of inflammation in patients with quiescent inflammatory bowel disease in a randomized trial. Gastroenterology.

[16] Cox SR, Czuber-Dochan W, Wall CL (2022). Improving food-related quality of life in inflammatory bowel disease through a novel web resource: a feasibility randomised controlled trial. Nutrients.

[17] Fritsch J, Garces L, Quintero MA (2021). Low-fat, high-fiber diet reduces markers of inflammation and dysbiosis and improves quality of life in patients with ulcerative colitis. Clin Gastroenterol Hepatol.

[18] Gash KJ, Goede AC, Chambers W, Greenslade GL, Dixon AR (2011). Laparoendoscopic single-site surgery is feasible in complex colorectal resections and could enable day case colectomy. Surg Endosc.

[19] Grimstad T, Berge RK, Bohov P (2011). Salmon diet in patients with active ulcerative colitis reduced the simple clinical colitis activity index and increased the anti-inflammatory fatty acid index--a pilot study. Scand J Clin Lab Invest.

[20] James SL, Christophersen CT, Bird AR, Conlon MA, Rosella O, Gibson PR, Muir JG (2015). Abnormal fibre usage in UC in remission. Gut.

[21] Jia W, Whitehead RN, Griffiths L (2010). Is the abundance of Faecalibacterium prausnitzii relevant to Crohn's disease?. FEMS Microbiol Lett.

[22] Jian L, Anqi H, Gang L, Litian W, Yanyan X, Mengdi W, Tong L (2018). Food exclusion based on IgG antibodies alleviates symptoms in ulcerative colitis: a prospective study. Inflamm Bowel Dis.

[23] Krag A, Israelsen H, von Ryberg B, Andersen KK, Bendtsen F (2012). Safety and efficacy of Profermin® to induce remission in ulcerative colitis. World J Gastroenterol.

[24] Kyaw MH, Moshkovska T, Mayberry J (2014). A prospective, randomized, controlled, exploratory study of comprehensive dietary advice in ulcerative colitis: impact on disease activity and quality of life. Eur J Gastroenterol Hepatol.

[25] Limdi JK, Aggarwal D, McLaughlin JT (2016). Dietary practices and beliefs in patients with inflammatory bowel disease. Inflamm Bowel Dis.

[26] Melgaard D, Sørensen J, Riis J (2022). Efficacy of FODMAP elimination and subsequent blinded placebo-controlled provocations in a randomised controlled study in patients with ulcerative colitis in remission and symptoms of irritable bowel syndrome: a feasibility study. Nutrients.

[27] Morvaridi M, Jafarirad S, Seyedian SS, Alavinejad P, Cheraghian B (2020). The effects of extra virgin olive oil and canola oil on inflammatory markers and gastrointestinal symptoms in patients with ulcerative colitis. Eur J Clin Nutr.

[28] Racine A, Carbonnel F, Chan SS (2016). Dietary patterns and risk of inflammatory bowel disease in Europe: results from the EPIC study. Inflamm Bowel Dis.

[29] Sánchez-Morales A, Pérez-Ayala MF, Cruz-Martínez M (2019). [Probiotics’ effectiveness on symptoms, histological features and feeding tolerance in ulcerative colitis]. Rev Med Inst Mex Seguro Soc.

[30] Sitkin SI, Tkachenko EI, Vakhitov TIa, Oreshko LS, Zhigalova TN (2013). [Serum metabolome by gas chromatography-mass spectrometry (GC-MS) in patients with ulcerative colitis and celiac disease]. Eksp Klin Gastroenterol.

[31] Strisciuglio C, Giannetti E, Martinelli M, Sciorio E, Staiano A, Miele E (2013). Does cow's milk protein elimination diet have a role on induction and maintenance of remission in children with ulcerative colitis?. Acta Paediatr.

[32] Meeralam YK, Al Zanabgi A, Mosli M (2023). A regional survey of awareness of inflammatory bowel disease among the Saudi population. Inflamm Intest Dis.

[33] Napolitano D, Martella P, Schiavoni E (2020). The awareness of the IBD nurse position among patients from an Italian tertiary IBD centre. Prof Inferm.

[34] Ratajczak AE, Festa S, Aratari A, Papi C, Dobrowolska A, Krela-Kaźmierczak I (2022). Should the Mediterranean diet be recommended for inflammatory bowel diseases patients? A narrative review. Front Nutr.

[35] Parra RS, Chebli JM, Amarante HM (2019). Quality of life, work productivity impairment and healthcare resources in inflammatory bowel diseases in Brazil. World J Gastroenterol.

[36] Ronchetti C, Cirillo F, Di Segni N, Cristodoro M, Busnelli A, Levi-Setti PE (2022). Inflammatory Bowel Disease and Reproductive Health: From Fertility to Pregnancy-A Narrative Review. Nutrients.

[37] Teigen L, Biruete A, Khoruts A (2023). Impact of diet on hydrogen sulfide production: implications for gut health. Curr Opin Clin Nutr Metab Care.

[38] Gu B, Yu Z, Shi C, Yan C, Chen B, Zhou J (2022). Effects of low-FODMAP diet on irritable bowel symptoms in patients with quiescent inflammatory bowel disease: a protocol for a systematic review and meta-analysis. Medicine (Baltimore).

[39] Peng Z, Yi J, Liu X (2022). A low-FODMAP diet provides benefits for functional gastrointestinal symptoms but not for improving stool consistency and mucosal inflammation in IBD: a systematic review and meta-analysis. Nutrients.

[40] Zhong M, Sun Y, Wang HG, Marcella C, Cui BT, Miao YL, Zhang FM (2020). Awareness and attitude of fecal microbiota transplantation through transendoscopic enteral tubing among inflammatory bowel disease patients. World J Clin Cases.

[41] Rashed R, Valcheva R, Dieleman LA (2022). Manipulation of gut microbiota as a key target for Crohn's disease. Front Med (Lausanne).

[42] Mcilroy JR, Nalagatla N, Hansen R, Hart A, Hold GL (2018). Faecal microbiota transplantation as a treatment for inflammatory bowel disease: a national survey of adult and paediatric gastroenterologists in the UK. Frontline Gastroenterol.

[43] Jiang S, Miao Z (2023). High-fat diet induces intestinal mucosal barrier dysfunction in ulcerative colitis: emerging mechanisms and dietary intervention perspective. Am J Transl Res.

[44] Sinopoulou V, Gordon M, Dovey TM, Akobeng AK (2021). Interventions for the management of abdominal pain in ulcerative colitis. Cochrane Database Syst Rev.

[45] Yao CK, Sarbagili-Shabat C (2023). Gaseous metabolites as therapeutic targets in ulcerative colitis. World J Gastroenterol.

[46] Teigen LM, Geng Z, Sadowsky MJ, Vaughn BP, Hamilton MJ, Khoruts A (2019). Dietary factors in sulfur metabolism and pathogenesis of ulcerative colitis. Nutrients.

[47] Radziszewska M, Smarkusz-Zarzecka J, Ostrowska L, Pogodziński D (2022). Nutrition and supplementation in ulcerative colitis. Nutrients.

[48] LeBlanc JF, Segal JP, de Campos Braz LM, Hart AL (2021). The microbiome as a therapy in pouchitis and ulcerative colitis. Nutrients.

